# Sustained Release of Antifibrotic Nintedanib from Polymer Microparticles Reduces Dosing Frequency While Reducing Inflammation in Murine Idiopathic Pulmonary Fibrosis

**DOI:** 10.1007/s10439-025-03729-8

**Published:** 2025-04-10

**Authors:** Emmanuel Einyat Opolot, Filip Goshevski, Rahul Chaudhary, Jessica A. Kilgore, Noelle S. Williams, Horst A. von Recum, Amar B. Desai

**Affiliations:** 1 Department of Biomedical Engineering, Case Western Reserve University, Cleveland, OH USA; 2 Department of Medicine and Case Comprehensive Cancer Center, Case Western Reserve University, Cleveland, OH USA; 3 Department of Biochemistry, University of Texas Southwestern Medical Center, Dallas, TX USA

**Keywords:** Nintedanib, Idiopathic pulmonary fibrosis, Polymer, Microparticles, Antifibrotic, Beta-cyclodextrin

## Abstract

**Purpose:**

Idiopathic pulmonary fibrosis (IPF) is a life-threatening, progressive lung disease with limited therapeutic options, often resulting in poor patient outcomes. Current treatments, such as Nintedanib (NTB) and Pirfenidone (PFD), require frequent administration, leading to adverse effects and low patient adherence. The purpose of this study was to investigate a sustained-release drug delivery system utilizing microparticles (MPs) composed of insoluble beta-cyclodextrin (β-CD) polymers to enhance the bioavailability and extend the release of NTB and PFD.

**Methods:**

A multidisciplinary approach, including in silico modeling, in vitro assays, and in vivo studies, was employed to assess the efficacy of β-CD-polymer MPs as drug carriers.

**Results:**

Molecular docking simulations and surface plasmon resonance studies demonstrated a stronger binding affinity of NTB to β-CD-polymer MPs compared to PFD, suggesting an extended delivery profile for NTB over PFD. Pharmacokinetic analysis in healthy mice confirmed sustained-release profiles for both drugs, with NTB maintaining therapeutic plasma concentrations for over 70 h. In a bleomycin-induced IPF mouse model, NTB-loaded β-CD-polymer MPs significantly reduced pro-inflammatory markers and required fewer injections than the standard daily NTB regimen.

**Conclusion:**

These findings indicate that β-CD-polymer MPs may serve as a promising platform for reducing dosing frequency of NTB and enhancing therapeutic outcomes in the treatment of IPF.

**Supplementary Information:**

The online version contains supplementary material available at 10.1007/s10439-025-03729-8.

## Introduction

Idiopathic pulmonary fibrosis (IPF) is a progressive, often fatal lung disorder affecting ~ 3 per 10,000 in North America [1]. The precise cause of IPF remains unknown [2]; however, it is an age-dependent disease whose prevalence is projected to rise significantly due to the predicted three-fold increase in the global elderly population by 2050 [3]. Thus, IPF poses a growing challenge to healthcare systems worldwide.

IPF is characterized by an excessive accumulation of extracellular matrix (ECM) and irregular lung healing, which leads to scarring, alteration of the alveolar structure, and irreparable loss of lung function. Additionally, IPF is often associated with other chronic lung diseases, including tuberculosis (TB) [4]. Individuals with IPF are at least six times more likely to develop TB [5], a leading cause of infectious disease mortality worldwide and a significant risk for immunocompromised patients [6]. Poor adherence to prescribed therapies in chronic diseases further complicates IPF management, with over 45% patients not following treatment regimen [7]. This non-adherence can exacerbate chronic infections like TB, advancing to a fibrotic state that perpetuates worsening disease, impaired pulmonary function, respiratory failure, and death [6]. This highlights a critical need for novel antifibrotic therapies that improve patient compliance and more effectively management of IPF.

There is currently no cure for IPF [8, 9], but treatment strategies have evolved to include oral medications, oxygen therapy, and in specific instances, surgical intervention to remove damaged lung tissue. A significant advancement has been the development of antifibrotic agents like Pirfenidone (PFD) and Nintedanib (NTB), which are among the few approved treatments for IPF. NTB works by inhibiting multiple tyrosine kinase signaling pathways, reducing cellular proliferation, migration, and extracellular matrix formation, thereby slowing disease progression [10]. Conversely, PFD works as an inflammatory modulator, primarily blocking the transforming growth factor (TGF-β) pathways to slow the thickening and stiffening of lung tissue, thus reducing respiratory difficulties in IPF patients [11]. However, the pharmacokinetic properties of these antifibrotic agents present challenges to their effective use. Limited bioavailability [12] and the need for frequent dosing due to the short half-life [13, 14] of the drugs contribute to systemic side effects [15]. These effects, along with required lifestyle changes like strict dieting and cessation of alcohol and smoking, often lead to patient non-adherence to treatment.

To address the challenges of patient compliance/adherence and treatment efficacy, recent research has focused on developing sustained drug delivery systems that enhance therapeutic effectiveness by facilitating localized delivery and extending drug bioavailability [16–21]. Insoluble cyclodextrin (CD) polymers have emerged as a promising strategy for drug delivery due to their ability to prolong drug release through the formation of stable CD/drug complexes. Specifically, microparticles (MPs) composed of insoluble beta-cyclodextrin (β-CD) polymers have been investigated for their potential to deliver antifibrotic drugs [15]. β-CD microparticles, distinguished by their ease of fabrication, tunable mechanical properties, and established in vivo biocompatibility and non-toxicity, stand out as a vehicle for controlled drug delivery. Furthermore, studies have shown that soluble cyclodextrin monomers can enhance drug solubility and bioavailability through complexation [22, 23]. The unique capability of cyclodextrins to form inclusion complexes with various drugs and other biomolecules makes them ideal candidates for controlled-release systems and sustained, long-term drug administration across a range of applications.

In this study, we present a novel approach for the controlled release of antifibrotic agents, Nintedanib (NTB) and Pirfenidone (PFD), loaded within insoluble β-CD-polymer MPs, to achieve targeted therapeutic effects in pulmonary tissue. Our objective is to develop a treatment regimen that is both more effective and more conducive to patient compliance by leveraging the physicochemical properties of antifibrotic drugs in combination with the advantages of polymeric carriers. This strategy is anticipated to provide prolonged, sustained, and localized delivery of these agents, which is critical for improving therapeutic outcomes in IPF patients and potentially mitigating the risk of secondary infections, such as tuberculosis.

## Materials and Methods

### Computer Simulated “Affinity” Predictions: Cyclodextrin—Nintedanib, Pirfenidone

To predict the binding affinity of NTB and PFD with cyclodextrin variants, and thereby developing an understanding of the potential for the insoluble CD-polymer to provide sustained delivery, we performed in silico docking simulations using PyRx v.0.9.7, (Molecular Graphics Labaratory, The Scripps Research Institute, La Jolla, CA, USA) a docking algorithm software and a Python-based affinity predictor, as described previously [15]. The chemical structures of NTB (CID: 135423438) and PFD (CID: 40632) were obtained from the PubChem database, while the structure beta (CID: 444041) cyclodextrin (β -CD) was sourced from the Protein Data Bank. All chemical structures were converted to and used in PDBQT format.

### Surface Plasmon Resonance: Beta Cyclodextrin—Nintedanib, Pirfenidone

The real-time interaction and binding affinity between NTB, PFD, and a β-CD monomer were also empirically observed using a Biacore X100 machine (GE Healthcare Bio-Sciences, Pittsburg, PA, USA) based on surface plasmon resonance (SPR). Using previously established cyclodextrin conjugation protocols [24], we prepared the CD-conjugated sensor surfaces for interaction with NTB and PFD. In brief, CM-3 chips were modified where the carboxymethyl cellulose surface was treated with EDC-NHS chemistry [25] where (3-dimethylaminopropyl)-ethylcarbodiimide (EDC), and N-hydroxysuccinimide (NHS) activate the carboxyl surface for reaction. The ligand 6-amino-6-deoxy β-cyclodextrin was then covalently conjugated to the carboxyl surface in a HEPES balanced salt buffer at a slightly basic pH of 7.4 and immediately followed by a surface deactivation with ethanolamine solution. For the assessment of the binding affinity, we ran a kinetic multicycle experiment for each of the analyte solutions NTB, and PFD, respectively. The running buffer for both NTB and PFD was phosphate-buffered saline. A 50 mM solution of sodium hydroxide was used to remove residual analyte between samples, for regeneration of the surface. The experimental data were then evaluated by fitting the steady-state affinity model. As specified in the equipment manual, a good fit was satisfied based on the scale of the Chi^2^(RU^2^) < 10.

### Synthesis and Characterization of Cyclodextrin Microparticles

For manufacture of the insoluble cyclodextrin polymers, a lightly crosslinked prepolymer of epichlorohydrin-cyclodextrin (CTD) (purchased from CycloLab in Budapest, Hungary) was dissolved in 200 mM (25%w/v) potassium hydroxide and heated for 10 min to a temperature of 60 °C. Tween85/Span85 solution (24%/76%) was added to a beaker of light mineral oil to warm it while being swirled at 500 rpm. Dropwise additions of ethylene-glycol-diglycidyl ether (EDGE) were made. A 2-min solution vortex was done and promptly followed with the addition of the crosslinker. Throughout the 3-h crosslinking procedure, the temperature was maintained at 70 °C and the mixing speed kept at 650 rpm. The microparticles were separated from the oil mixture by centrifugation at 200 × g, and then they were washed twice each time, first with excess hexanes, followed by acetone, and finally with deionized water (diH_2_O). Finally, diH2O was used to resuspend the microparticles before they were frozen and lyophilized for 3 days. The microparticle's shape and size were determined by scanning electron microscope (Thermo Scientific Apreo 2S) at the Swagelok Center for Surface Analysis of Materials at CWRU. The stability of the microparticles was determined through surface charge analysis using Zetasizer Ultra instrument (Malvern, model ZSU5700)

### β-CD MPs Loading and Release Protocol with Nintedanib and Pirfenidone

To load NTB and PFD into β-CD-polymer MPs, 20 mg of dried microparticles was suspended in 800 or 500 µL of 20 mg/ml concentrations of both drugs in DMSO. The MPs and the drugs were allowed to mix for 1.5 h. in an end-to-end mixer. 200 µL of diH_2_O was then added to the mixture and allowed to mix for 0.5 h. The MPs were vortexed, incubated for 48 h. at 4 °C, centrifuged at 10 K rpm for 3 min, and washed twice with sterile PBS. For in vitro release, drug-loaded β-CD MPs were mixed with a buffer (0.01 M PBS) and agitated with rotary shaking incubation at 37 °C. At daily time points, the β-CD MPs were centrifuged followed by a partial refill buffer exchange. NTB and PFD concentrations were measured in a quartz-glass-uv-96-well plate using a Microplate Reader (Multi-mode H1 Hybrid, BioTek Instruments). The detection wavelengths were 330 and 260 nm for NTB and PFD, respectively. The Loading efficiency was approximated and calculated as a ratio of the mass of drug released to mass of total drug used for loading the β-CD MPs.

### Mass Spectroscopy Analysis of In vivo Release Kinetics for NTB and PFD-loaded β-CD MPs

Animals were housed in the AAALAC-accredited facilities of the CWRU School of Medicine. The Case Western Reserve University Institutional Animal Care and Use Committee (IACUC) approved the husbandry and experimental procedures in accordance with approved IACUC protocols 2019-0065. The quantification of the delivery of NTB and PFD was evaluated in vivo, using a healthy mouse model. Male, C57BL/6 8-week-old mice received dosages of NTB and PFD-loaded β-CD-polymer MPs (8 and 6 mg in 200 μL of PBS, respectively) delivered by intraperitoneal injection, and peripheral blood was collected into serum-separator microtainer tubes (Becton-Dickinson) at elected time points (*n* = 5 mice per arm; 0.5, 2, 4, 24, 48, and 72, h. post-drug-administration). Serum (20 µL) was collected and stored in a freezer (−80 °C) prior to LC-MS/MS analysis of the samples at the Williams biochemistry lab at University of Texas Southwestern. For LC-MS analysis, A Sciex 4500 mass spectrometer was used (multiple-reaction monitoring mode) with an Agilent C18 XBD column, (5 μ packing 50 × 4.6 mm size). The mobile phases were A (water + 0.1% formic acid), and B (methanol + 0.1% formic acid). The liquid chromatography conditions included a flow rate kept constant at 1.5 mL/min and a typical gradient elution process was run—where mobile phases were repeatedly run from 3 to 100% to elute the compounds of interest for a total of 4.5 min per run.

### Drug Efficacy and Sustained-Release Study in an In Vivo Fibrosis Disease Model

A murine model of IPF was used in the evaluation of the efficacy of the sustained delivery of NTB in reducing disease severity. 9-week-old C57BL/6 mice were administered 1 mg/kg bleomycin via intratracheal instillation. From disease induction, the mice received intraperitoneal injections of 30 mg/kg of NTB twice-daily from day 0 to day 7 (d0–d7) or intraperitoneal injections of 8 mg/kg NTB-loaded β-CD-polymer MPs once on d0 and d4. Disease symptoms such as weight loss were recorded daily and blood serum was collected at harvest. The mice were sacrificed on day 7 post-injury. Serum samples were sent to Eve Technologies (A Canadian company which specializes in advanced multiplex assay technology for biomedical research and clinical diagnostics) to run their 32-plex mouse discovery assay. The same murine model (C57BL/6) infected with bleomycin (1 mg/kg) via intratracheal instillation was used to evaluate the effectiveness of only two administrations (d1 and d4) of NTB-loaded β-CD MPs in sustaining detectable levels of NTB in an IPF disease model for a 7-day study period.

### β-CD MPs Toxicity Study

A healthy male murine model was used to investigate the safety of β-CD MPs in vivo. 8-week-old C57BL/6 mice were administered with both drug (NTB, 8 mg/kg) loaded, and non-drug-loaded β-CD MPs administered for 15 days. Weight loss was recorded on days d1, d4, d7, d10, d13, and d15 to assess animal health. Complete blood counts (CBCs) were collected on d15, as well as serum for a chemistry panel.

### Statistical Analysis

Except where otherwise noted, all experiments were done in triplicate, and data were provided as the mean plus standard error. Error bars represent the standard deviation from the arithmetic mean. In vivo data for mice are expressed as the average of *n* = 5.

## Results

### Predicted Affinity of β-CD for Nintedanib Compared to Pirfenidone

**Fig. 1:**
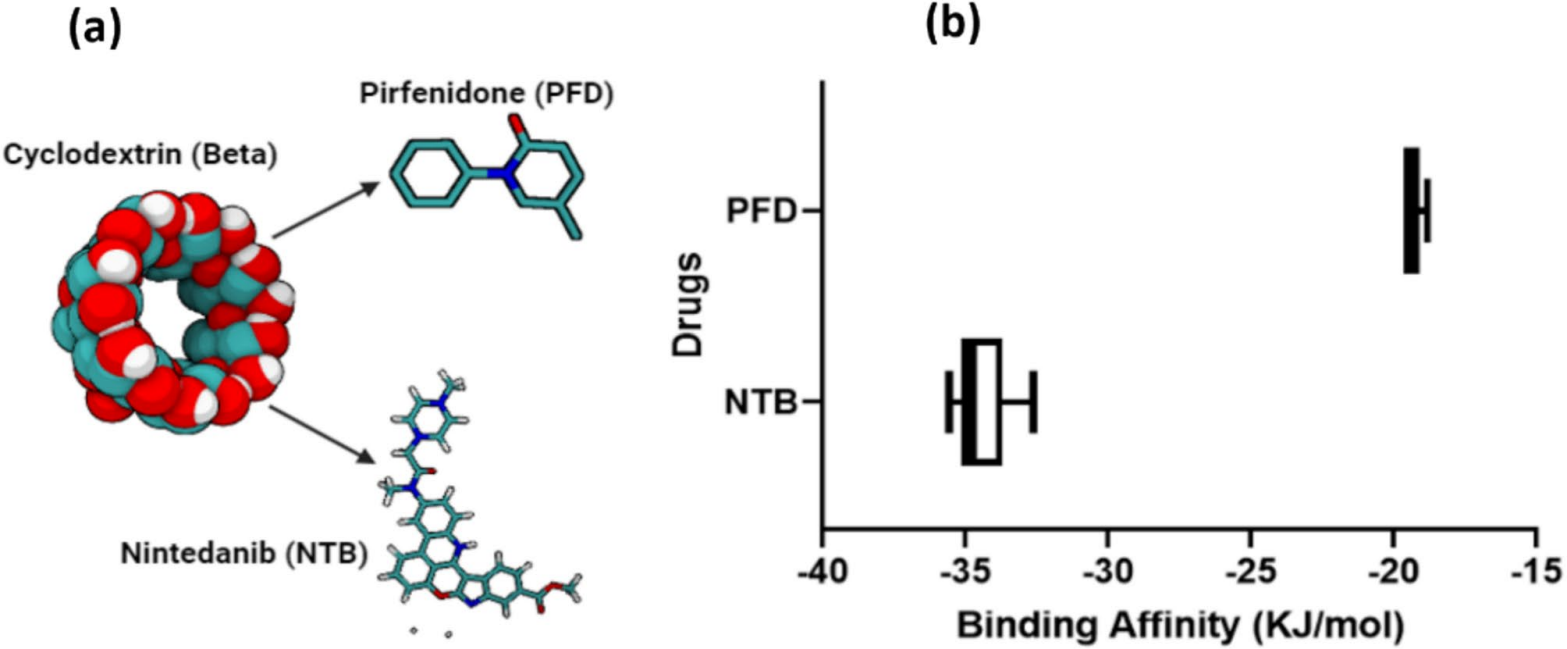
In silico simulation of binding affinity: **a** Molecular interaction models of β-cyclodextrin with antifibrotic agents. The inclusion complex of β-cyclodextrin is visualized in a 3D molecular form, demonstrating potential host–guest interactions with Pirfenidone (PFD) and Nintedanib (NTB). Structural formulas of PFD and NTB are provided alongside their respective molecular conformations within the β-cyclodextrin cavity. **b** Quantitative Assessment of Binding Affinities. The bar graph presents the binding affinities of PFD and NTB to β-cyclodextrin, measured in kilojoules per mole (KJ/mol). The data points represent the mean values of binding energies, with the error bars indicating the standard deviation (*n* = 8)

We first evaluated the binding affinity of NTB and PFD to β-CD using a docking software application (PyRx). We observed that NTB has a stronger predicted binding affinity for β-CD compared to PFD, with binding energies of − 34.9 KJmol^-1^ vs − 19.3 KJmol^-1^ (Fig. 1B). This suggests a greater propensity for sustained delivery from insoluble β-CD-polymers for NTB over PFD.

### Surface Plasmon Resonance

To validate the predicted binding affinities, we performed surface plasmon resonance (SPR) experiments. NTB and PFD were flowed over a sensor chip conjugated with a 6-amino-6-deoxy-β-CD ligand. Analysis of the sensorgrams using a steady-state affinity model revealed that NTB exhibited a higher binding affinity (3.8 × 10^-8^ M) compared to PFD (1.7 × 10^2^ M) as shown in Table 1. These findings are consistent with the predicted affinities obtained from the docking simulations in Fig. 1.

**Table 1:**
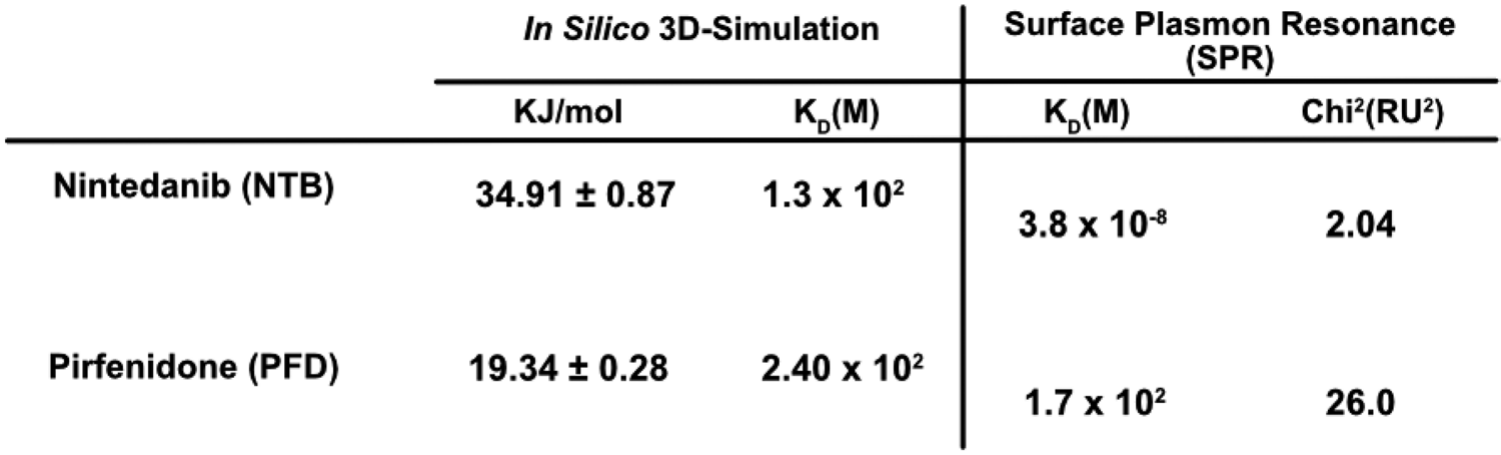
Analysis of binding affinities and molecular interactions between beta cyclodextrin and two analytes, Nintedanib (NTB) and Pirfenidone (PFD): the equilibrium dissociation constants (KD) and goodness-of-fit (Chi^2^(RU^2^)) obtained from SPR experiments using a 1:1 steady-state binding model, alongside calculated binding energies from in silico molecular dynamics simulations

### Characterization of β-CD MPs: Size, and Zeta Potential of β-CD MPs

**Fig. 2:**
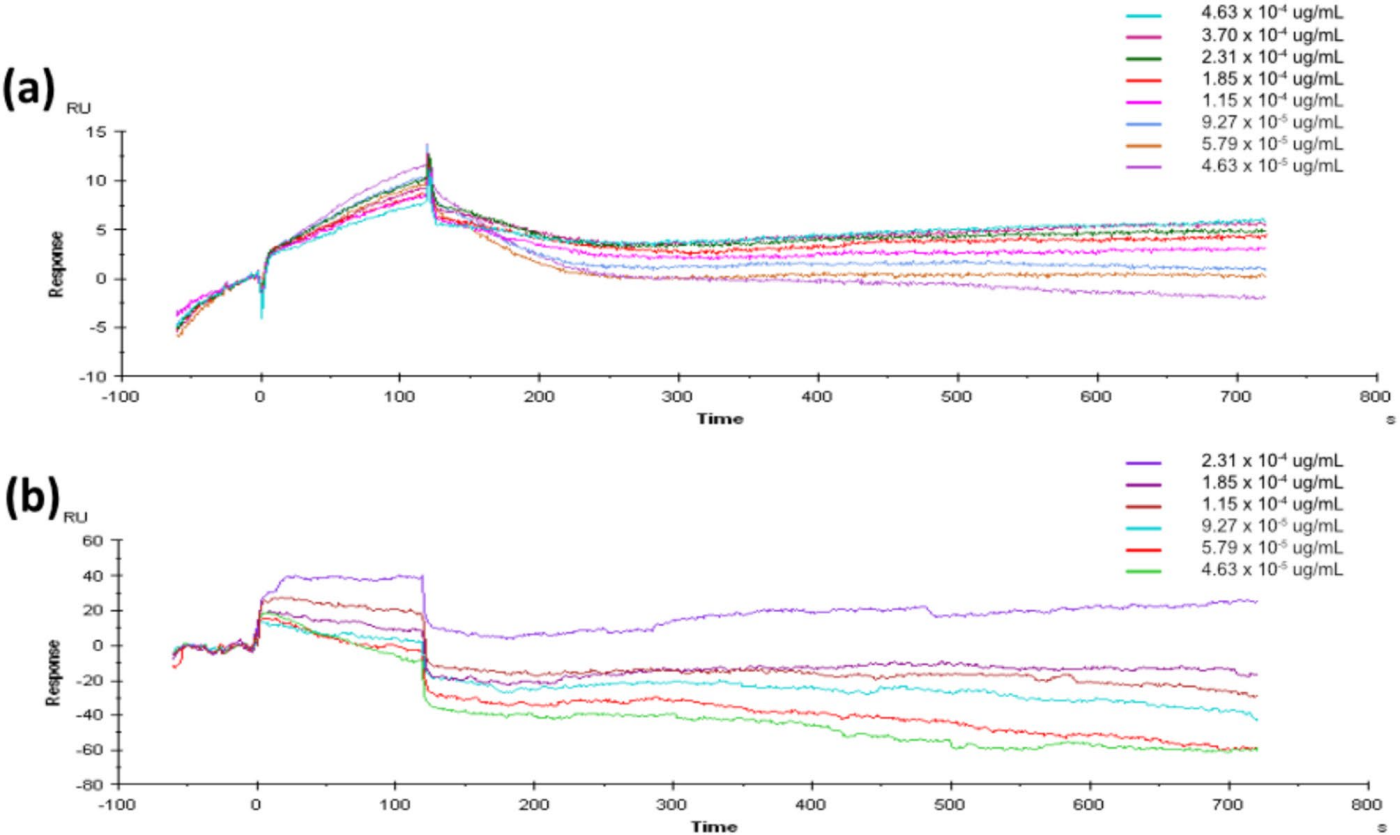
Analysis of binding affinities and molecular interactions between beta cyclodextrin and two analytes, Nintedanib (NTB) and Pirfenidone (PFD), Using Surface Plasmon Resonance (SPR) and in silico 3D-Simulation Techniques: **a** Sensorgram for NTB, **b** Sensorgram for PFD. The sensorgrams illustrate real-time binding interactions over the course of the experiment. The SPR data indicate a higher binding affinity of NTB to beta cyclodextrin compared to PFD, which is corroborated by the calculated binding energy from the 3D simulations

To determine the morphology and size distribution of the β-CD MPs after synthesis, scanning electron microscopy was used. The β-CD MPs exhibited a spherical morphology with smooth surfaces and were 28.03 ± 6.84 µm in average size (Fig. 2a and b) suggesting successful fabrication and a controlled size distribution which are essential factors for drug encapsulation and controlled-release applications [26]. The surface charge of the β-CD MPs was assessed via zeta potential measurements (Fig. 2d) of a − 9.67 ± 1.28 mV average [27].

### β-CD MPs Prolong Nintedanib Release In Vitro

To evaluate the in vitro drug release from the β-CD-polymer MPs, NTB and PFD were loaded with final concentrations of 16 mg/mL and incubated at 4 °C for 48 h. as outlined in the loading protocol, recovered and washed twice by centrifugation before mixing with the release buffer (0.01 M PBS), and agitated on an end-to-end mixer. At predetermined intervals of at least 24 h, the β-CD MPs were centrifuged at 10 k rpm for 180 s and a partial amount of release buffer was aliquoted and replaced with an equal amount of fresh release buffer. Consistent with our prediction, the β-CD MPs maintained steady cumulative release for both drugs with PFD showing a burst release within the first 144 h. and plateaus at 2.43 mg/mL by 216 h. while NTB continues to release for at least 400 h. reaching 4.65 mg/mL (Fig. 2c). A statistical analysis using an independent *t*-test revealed no significant difference in NTB and PFD release within the first 24 h (*p* > 0.05). However, from 48 to 120 h, significant differences emerged (*p* < 0.05), with PFD reaching a plateau between 144 and 216 h, while NTB continued to increase. From 240 to 384 h, highly significant differences were observed (*p* < 0.001), indicating a sustained-release profile for NTB compared to PFD. This sustained release is likely due to the affinity interactions. Moreover, the calculated loading efficiency was 46.5% for NTB and 24.3% for PFD.

### β-CD-polymer Microparticles Enable Prolonged Release of Nintedanib

**Fig. 3:**
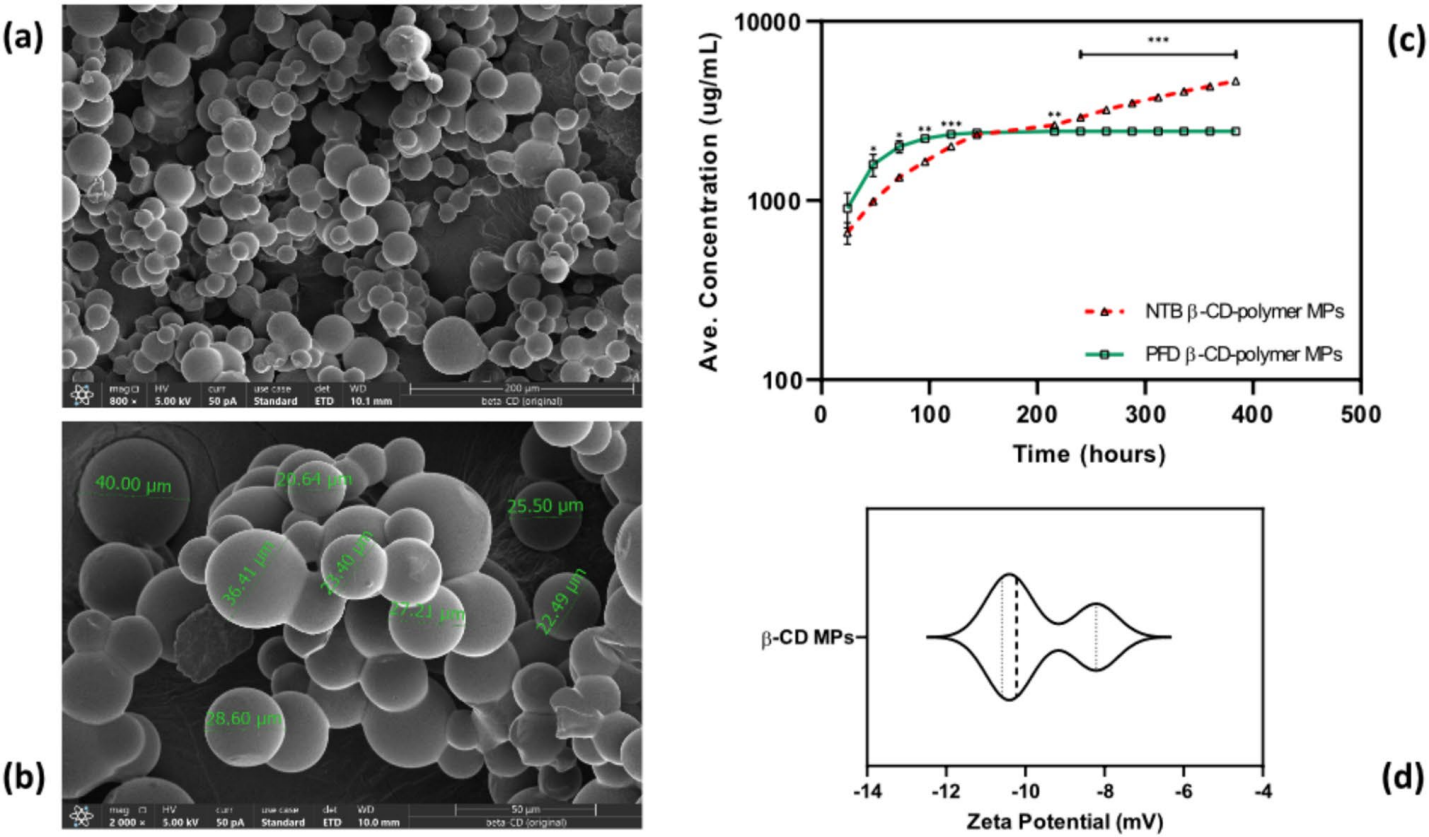
Characterization of β-CD MPs: **a, b** Scanning electron microscopy (SEM) images of β-CD MPs at different magnifications, showing their spherical morphology and size distribution. **c** Cumulative in vitro drug-release profiles of NTB and PFD β-CD-polymer MPs over time, indicating sustained-release behavior. **d** Zeta potential analysis of β-CD MPs, demonstrating their surface charge properties

To evaluate the prolonged release of NTB and PFD from insoluble β-CD-polymer MPs, we administered drug-loaded microparticles to healthy mice via intraperitoneal injection and compared them to bolus intraperitoneal injections of NTB and PFD. Serum was collected from the mice at intervals of 0.5, 24, 48, and 72 h post-administration to quantify drug levels. NTB-loaded β-CD-polymer particles demonstrated an expected initial burst release, followed by a sustained plateau over 72 h. In contrast, the PFD-loaded β-CD-polymer particles exhibited an expected more-rapid delivery over the same period. Both NTB and PFD administered as 10 mg/kg bolus injections showed a rapid decline in bioavailability within the first 24 h with the 10 mg/kg PFD bolus injection having a higher initial peak (Fig. 3).

### β-CD MPs Maintain Detectable NTB Concentration In Vivo in IPF Disease Model

**Fig. 4:**
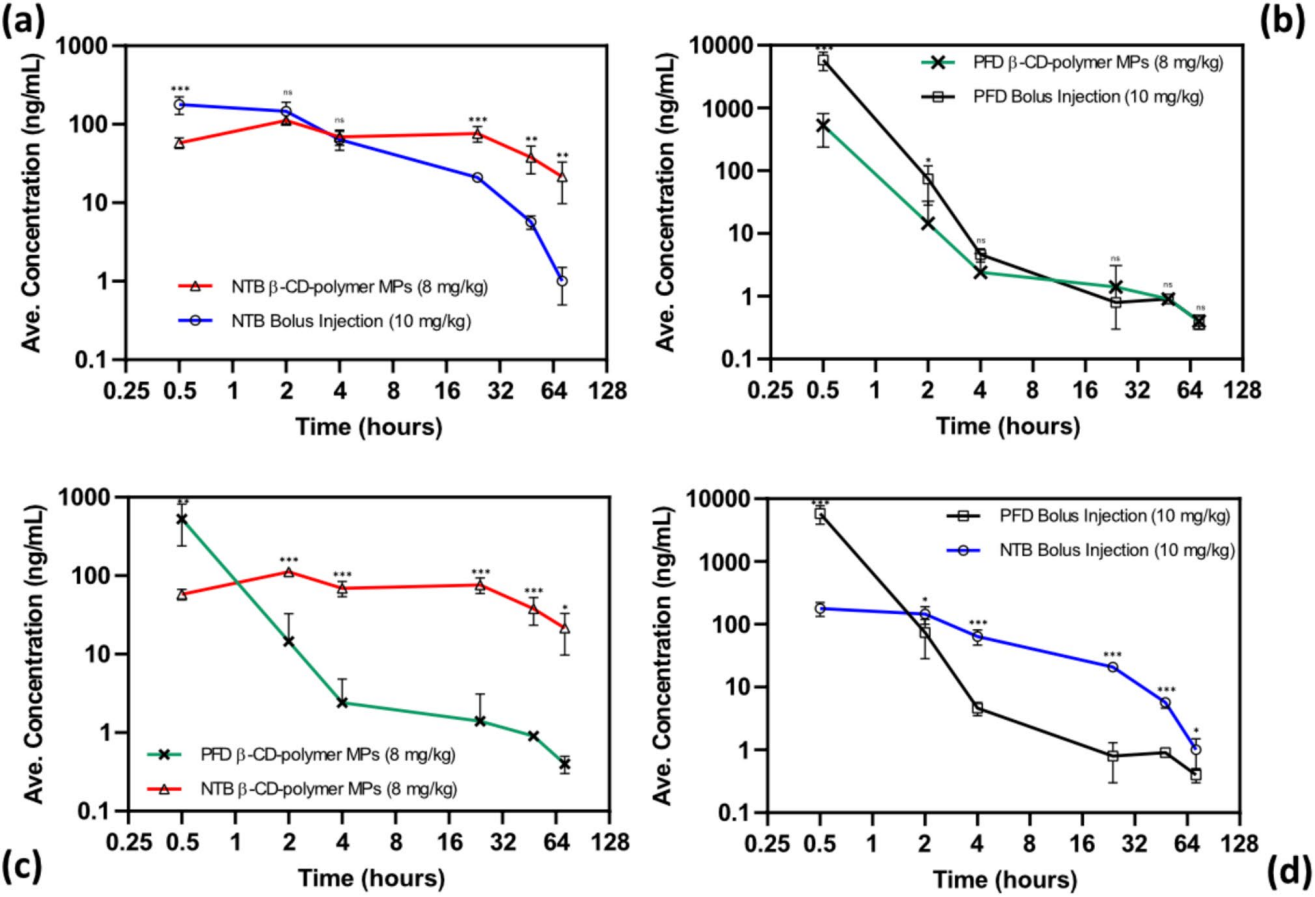
Comparison of NTB vs PFD in vivo tolerance study of serum-level profiles of drug-loaded β-CD-polymer MPs and free drugs over time: the graph outlines the average compound concentration (ng/mL) in plasma following intraperitoneal administration of drug-loaded β-CD-polymer MPs and free drug injections. The profiles are plotted over a period of 72 h post-administration. Serum NTB and Pirfenidone concentrations collected from mice (*n* = 5 at each time point) following intraperitoneal administration. **a** NTB β-CD-polymer MPs (8 mg/kg) vs. NTB bolus injection (10 mg/kg), showing sustained release from microparticles. **b** PFD β-CD-polymer MPs (8 mg/kg) vs. PFD bolus injection (10 mg/kg), demonstrating rapid clearance of both formulations. **c** Direct comparison of NTB and PFD β-CD-polymer MPs, highlighting prolonged NTB retention. **d** NTB vs. PFD bolus injections, illustrating significantly higher initial PFD concentrations with rapid elimination. Data are presented as mean ± SD (*n* = 5), with statistical significance denoted (*p < 0.05, **p < 0.01, ***p < 0.001, ns = not significant, independent *t*-test)

To evaluate whether β-CD MPs can maintain detectable NTB levels in an IPF disease model, C57BL/6 mice were infected with bleomycin (1 mg/kg) via intratracheal instillation and treated with NTB-loaded β-CD MPs (administered on d1, and d4), and a PBS control (administered from d1 to d7). Serum was collected on d7 for LC-MS analysis and body weight was monitored throughout disease progression. Fig. 4a shows NTB serum levels at 300 ng/mL, indicating presence of NTB post-treatment with NTB-loaded β-CD MPs in an IPF disease state over the course of the study Fig. 4b shows percent weight change over 7 days where significant differences (*p < 0.05, Wilcoxon signed-rank test and paired t-test) were noted between the treatment groups. Treatment with the NTB-loaded β-CD MPs shows the lowest average weight loss, suggesting some protection against weight loss during disease progression.

### Sustained NTB Delivery Alleviates IPF Symptoms

To assess the therapeutic efficacy of sustained NTB delivery in a disease model, we used the bleomycin-induced murine model of IPF [28]. C57BL/6 mice were infected with bleomycin (1 mg/kg) via intratracheal instillation and treated with bolus NTB (twice-daily from d0 to d7) and NTB-loaded β-CD-polymer MPs (d0 and d4 only) via intraperitoneal injections. Body weight was monitored as a key indicator of disease progression and overall health following disease induction and treatment with either bolus NTB or NTB-loaded β-CD-polymer MPs. Fig. 4 shows the percent weight loss, normalized to the initial weight, over the course of the study. The untreated (Bleo) group exhibited the most severe weight loss, indicating successful disease induction. Both NTB treatments, particularly the bolus NTB, mitigated weight loss compared to the untreated group, suggesting a protective effect that against bleomycin-induced weight loss. However, neither treatment completely prevented weight loss (Fig. 4).

### No Observable Toxicity from β-CD MPs—with or without Drug

The safety assessment of NTB-loaded microparticles (β-CD NTB(Bleo)) in a healthy mouse model for 15 days, revealed no observable toxicity based on the complete blood count (CBCs) analyses (Fig. 7a–d) and serum chemistry. (Fig. 7e–j) No significant hematologic abnormalities were detected, aside from a mild increase in lymphocytes in the NTB B-CD group, and liver and kidney function markers remained unchanged across groups. A slight but non-significant reduction in body weight was observed in both microparticle-treated groups compared to naïve control. Additionally, no difference was found between empty and NTB-loaded β-CD MPs, suggesting the effect is likely independent of NTB. (Fig. 7k) The absence of major adverse effects in biochemical and hematologic parameters indicates that NTB-loaded β-CD MPs are well tolerated for 15 days, with only mild systemic effects. These findings support the potential for further investigation of NTB-loaded β-CD MPs as a therapeutic strategy while emphasizing the need for additional studies to further explore the physiological impact of microparticles on weight regulation.

### Sustained Release of NTB Reduces Inflammatory Response in IPF

**Fig. 5:**
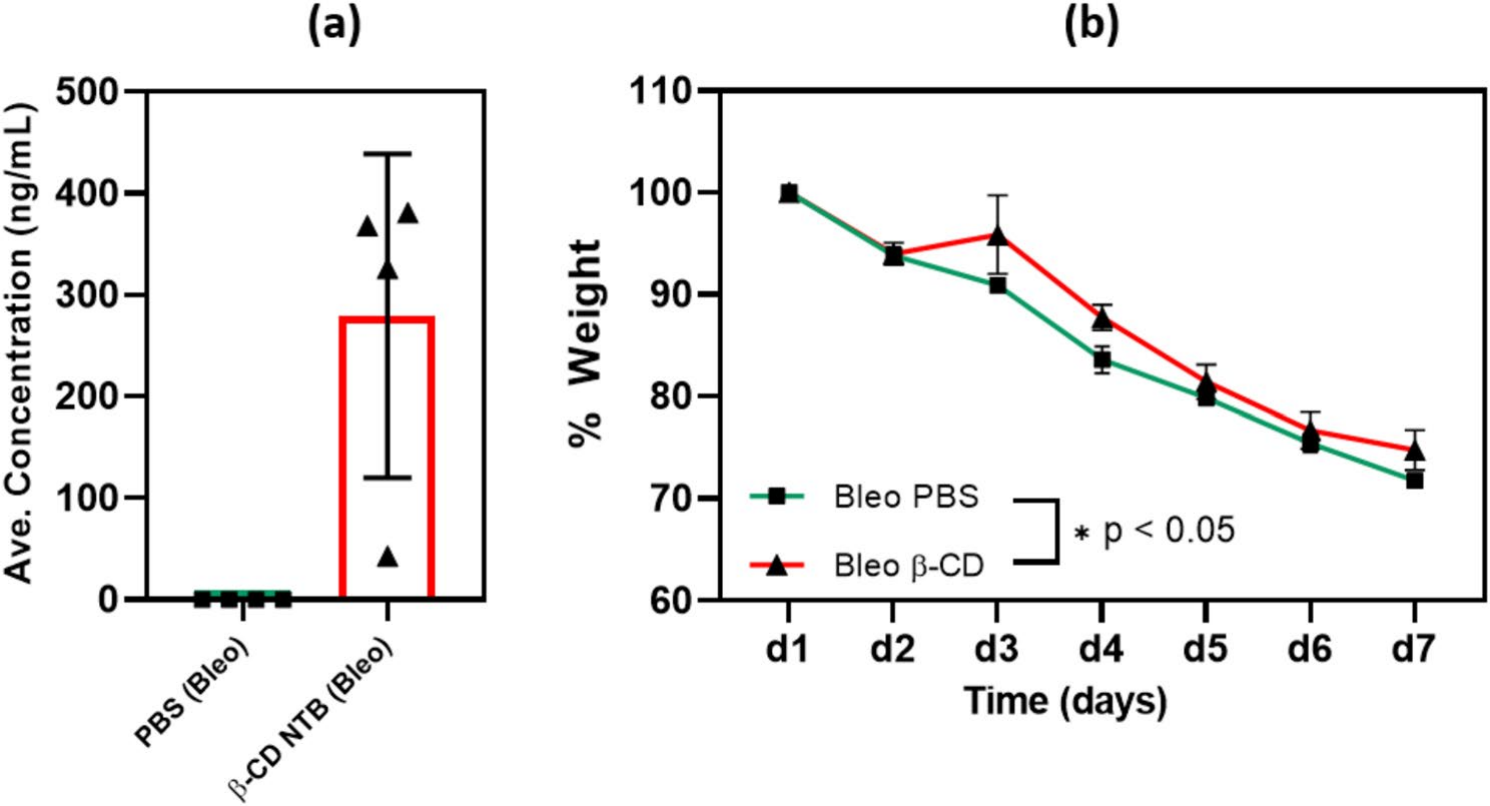
β-CD MPs maintain detectable systemic NTB levels and impact body weight in bleomycin-induced fibrosis. **a** Serum NTB concentrations detectable in serum after treatment with NTB-loaded β-CD MPs only twice in a 7-day study. Data shown as mean ± SD.** b** Percent body weight change over time for the control group (Bleo PBS) and the treated group (Bleo β-CD). A significant difference was observed (*p < 0.05, Wilcoxon signed-rank test and paired t-test), indicating the protective effect of NTB-loaded β-CD MPs treatment on weight loss

Serum cytokine and chemokine levels in the mice were determined to assess the impact of sustained NTB release on inflammation following IPF induction. Mice treated with NTB-loaded β-CD-polymer MPs showed significantly lower levels of Interferon gamma (IFNγ) and C-X-C motif chemokine 5 (LIX) compared to untreated controls, indicating a reduction in inflammatory response (Fig. 5a and b). Both NTB treatment groups, including those receiving NTB-loaded β-CD-polymer MPs and bolus NTB, exhibited similar reductions in profibrotic chemokines, such as CXCL10 (IP-10) and monocyte chemoattractant protein-1 (MCP-1), compared to elevated levels in the untreated Bleo only group (Fig. 5c and d). These findings suggest that sustained NTB delivery effectively reduces key inflammatory and profibrotic markers in the serum, contributing to its therapeutic potential in managing IPF.

## Discussion

The absence of a definitive cure for IPF remains a major concern for global healthcare [9], particularly given the disease’s low post-diagnosis survival rates and its increasing prevalence in the aging population [8, 29]. While the approval of antifibrotic medications like NTB and PFD represents an important step toward treating the disease, there remain hurdles to overcome. One hurdle is the high patient cost associated with these treatments- in the US, the average annual all-cause cost for a PFD treatment regimen is over $12,000 while NTB is approximately $20,000 per patient. IPF care alone exerts at least $1.5 billion in financial pressure to the economy [30, 31]. These economic and clinical challenges underscore the need for improved drug delivery systems that enhance bioavailability and reduce treatment frequency. This study introduces a novel approach which leverages polymer-based affinity binding to improve the delivery and bioavailability of NTB using β-CD-polymer MPs as a delivery vehicle for intraperitoneal delivery. Our findings suggest that β-CD-polymer MPs can decrease (1) the frequency of drug administrations and (2) the overall amount of drug required while maintaining therapeutic efficacy. This strategy addresses key challenges in IPF treatment, including patient compliance, frequent dosing requirements, and high cost of the treatment. Cyclodextrin-based delivery systems have previously been utilized to prolong the administration of various small-molecule therapeutics [15, 32]. Here, we report the first investigation of a CD-polymer as a long-term delivery mechanism for NTB. Cyclodextrins consist of glucose monomers uniquely arranged into a bucket-like toroid which features primary hydroxyl groups at the narrower end, and secondary hydroxyl groups along the broader rim. This frustrum-like structure confers cyclodextrin with a hydrophobic interior cavity and a hydrophilic exterior, a characteristic which is central to their functionality in host–guest interactions. These interactions facilitate the formation of inclusion complexes with various, usually, poorly water-soluble drugs and biomolecules. The binding mechanism within these complexes is affinity-based, driven by forces which include hydrophobic interactions, electrostatic charges, van der Waals forces, and ionic bonds. The guest molecule, when encapsulated within the cyclodextrin’s cavity, benefits from the hydrophilic shell of the host, enhancing its solubility in aqueous environments. This encapsulation significantly improves the guest molecule’s absorption and bioavailability [33, 34]. These affinity binding interactions hinge on the strength and specificity of the attraction between the host and the guest [35, 36]. Therefore, through computational analysis, we predicted strong binding affinity between NTB with β-CD (Fig. 1). The binding energy between NTB and β-CD is comparable to that of other drugs including rifampicin that have been shown to form stable complexes with β-CD [24]. This suggested that β-CD would form a stable complex with NTB to facilitate sustained release of the drug. Additionally, similar to work by Acipreste Hudson et al., [37] our SPR analysis compared the real-time interaction between β-CD and both PFD and NTB which confirmed that NTB is better suited for delivery (Table 1 and Fig. 2). The significant discrepancy between the in silico computed K_D_ and the experimentally determined K_D_ likely reflects limitations of the PyRx docking models as described in the study by Xin et al. [38].

We fabricated spherical β-CD MPs with smooth surfaces and an average size of 28.03 ± 6.84 µm (Fig. 3a and b) similar to studies by Rohner et al. [39] and Mohamed Omar et al. [40]. We investigated other characteristics of the β-CD MPs including the in vitro drug loading, zeta potential, and even calculated the loading efficiency for both NTB and PFD. As expected, the β-CD MPs prolonged the release of NTB to at least 400 h. In vitro in comparison with PFD (Fig. 3c) as predicted through both our in silico simulations (Fig. 1) and SPR analysis (Table 1, and Fig. 2) on the binding affinities of NTB and PFD to β-CD. The zeta potential was found to average approximately − 9.67 ± 1.28 mV (Fig. 3d) as the surface charge of the MPs which is similar to results from a study by Waqar et al. [27]. Although, with this zeta potential value, the β-CD MPs fall into a range indicative of limited stability [41], for our target application of sustained drug delivery using the β-CD MPs, this is likely advantageous as it reduces interaction with physiological membranes and thus reduces internalization of the MPs by cells as explained in a study by Öztürk et al. [42] For the calculated loading efficiency, NTB had a higher loading efficiency with 46.5% in comparison with 24.3% for PFD. These results fall within an acceptable range for moderately lipophilic drugs like NTB and PFD as explained in a study by Gadade et al. [43] and are aligned with the predicted binding affinity for NTB and PFD. Given these findings, further investigation into the release kinetics of NTB and PFD from the β-CD MPs was essential.

Understanding drug-release kinetics is crucial for developing effective delivery systems. These kinetics predict not only the behavior of the drug in clinical settings but also the dose consistency and safety from any adverse effects of that drug. It is essential that the kinetics of a drug’s release are evaluated in a controlled environment which is typically healthy animal models. In our study, we verified the sustained release of NTB from the β-CD-polymer MPs through in vivo tests in healthy mice. Our results demonstrate that higher levels of NTB released from the β-CD-polymer MPs were detectable in the blood serum for a significantly longer duration compared to PFD (Figs. 4a, c, and 5a). This prolonged presence of NTB in the blood serum supports our hypothesis that higher affinity for β-CD leads to slower release of the drug, thereby extending its bioavailability. This is similar to the findings in the study by Jermoskaite et al. [44]. Moreover, studies by Edgardo et al. [35] and Stella et al. [34] also explain that the main mechanism of drug release from CD-polymers involves dissociation through dilution—where the drug molecules are progressively released from the CD cavity as the guest–host complex interacts with a less concentrated environment, and the slow release is attributed to the sequential leaping of the drug molecules between binding sites within the inclusion complex matrix. Further, the bolus injection for PFD (Fig. 4b, and d) shows a high initial peak compared to that of NTB which is likely due to NTB having a lower solubility and not being easily absorbed [12, 45]. This high initial peak and subsequent rapid clearance of the injected PFD formulation enables high, transient systemic exposure which is useful for acute treatment modalities. For NTB, a drug with low solubility, its incorporation into β-CD pockets increases the bioavailability, resulting in prolonged release over 4 days. This suggests that β-CD-polymer MPs as a vehicle presents an opportunity to increase patient compliance by reducing the number of administrations of NTB, whose oral bioavailability remains low. The sustained-release profile is particularly beneficial for antifibrotic therapy in chronic diseases like IPF, where maintaining consistent dosing is important to effectively inhibit of disease progression [46]. Additionally, our results confirmed that β-CD-polymer MPs are well tolerated when administered through intraperitoneal injection, aligning with previous studies on the in vivo compatibility of cyclodextrin-based polymers. [47]

**Fig. 6:**
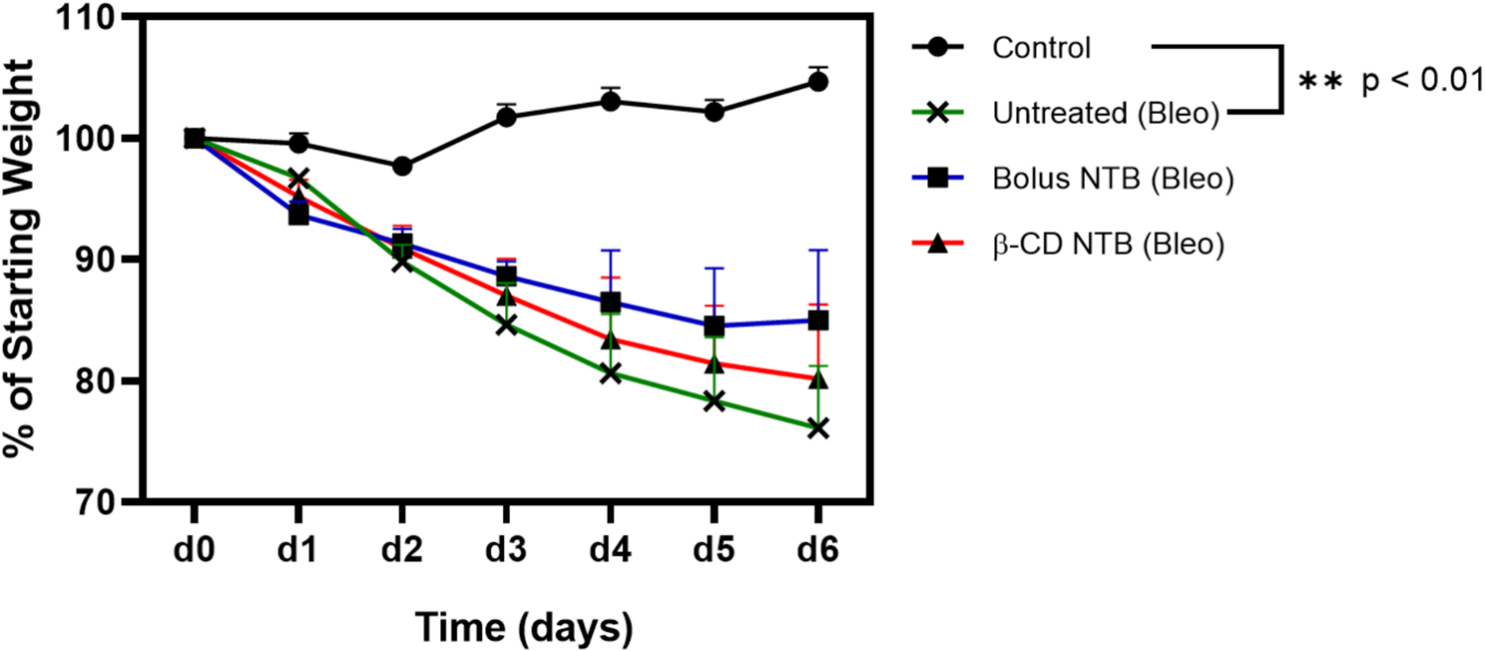
Percent Weight Loss During Disease Progression and Treatment: The graph shows the change in body weight (expressed as a percentage of initial weight over 6 days for the different experimental groups: Control, Untreated (Bleo), Bolus NTB, β-CD-polymer NTB (Bleo). The treatment groups exhibit weight loss. Although not significant, the Bolus NTB (Bleo) and β-CD- NTB (Bleo) groups show an improvement in the percent body weight lost in comparison with the Untreated (Bleo) group against the Control group (**p < 0.01, Dunnett’s test) with the largest decrease which suggests being most affected by the disease. Error bars represent the standard error of the mean (SEM) for each group at each time point. (*n* = 5 at each time point). Multiple comparisons ANOVA was performed for each condition

**Fig. 7:**
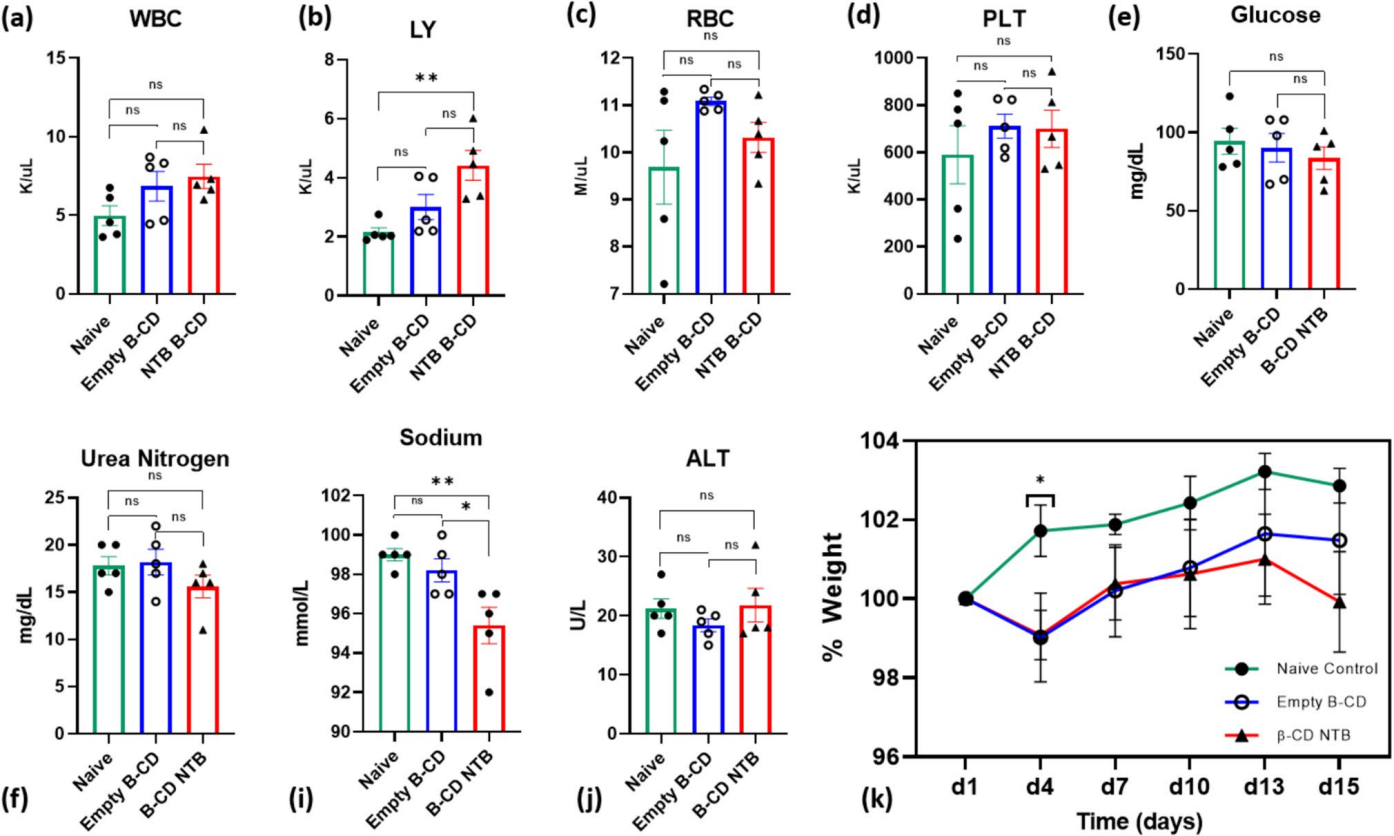
Comprehensive assessment of hematological, biochemical, and body weight changes following treatment with β-CD MPs. Hematological and serum chemistry analyses were performed to evaluate the safety of empty β-CD microparticles (empty β-CD) and NTB-loaded β-CD microparticles (β-CD NTB) in a bleomycin-induced fibrosis mouse model. **a–d** complete blood count (CBCs) results, including white blood cell count (WBC), lymphocytes (LY), red blood cell count (RBC), and platelet count (PLT). NTB-loaded β-CD MPs treatment significantly increased LY levels compared to the naïve group (*p* < 0.01), while other hematological parameters remained unchanged. **e–j** Serum chemistry analysis, including glucose, urea nitrogen, sodium, and alanine aminotransferase (ALT), showed no significant differences except for a decrease in sodium levels in the β-CD NTB group (*p* < 0.01, *p* < 0.05). **k** Body weight tracking over the 15-day study period revealed no significant differences between groups except for naïve control vs β-CD MPs on d4 (*p* < 0.05, Uncorrected Fisher’s LSD) indicating no adverse effects of the β-CD MPs. Data are presented as mean ± sem. (ns = not significant, *p < 0.05, **p < 0.01, Tukey’s post-hoc test) (SI-2) Additional hematological and serum analyses

**Fig. 8:**
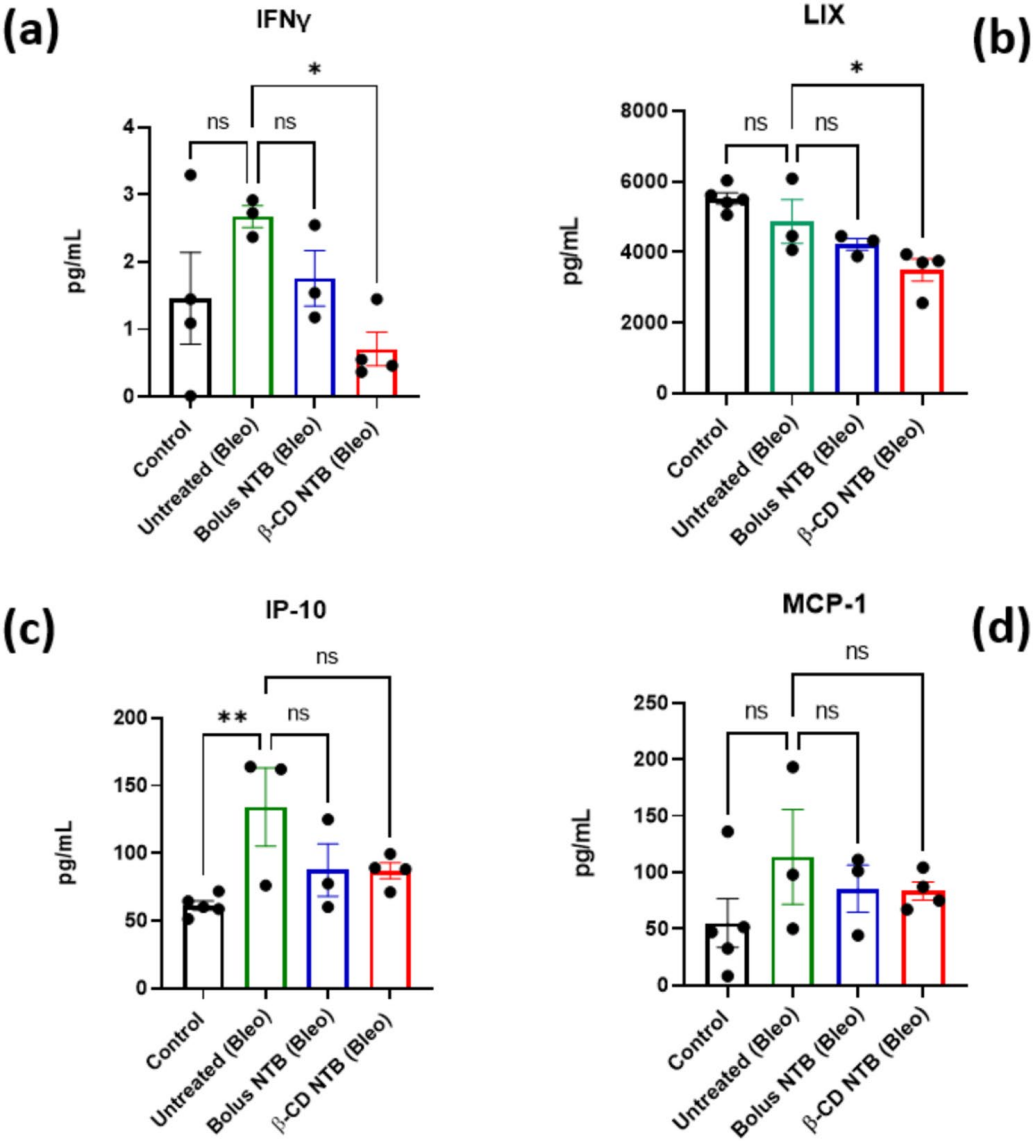
Multiplex ELISA analysis on serum cytokine/chemokine Levels: **a** IFNɣ, **b** LIX, **c** IP-10, and **d** MCP-1 levels were measured in the serum of mice. The experimental groups included the Control (PBS-treated), Untreated (Bleo) [Bleomycin-infected], Bolus NTB [bleomycin-infected, treated with free drug], and β-CD-polymer NTB [bleomycin-infected, treated with β-CD-polymer MPs loaded with NTB]. Significant reductions in IFNɣ and LIX levels were observed in the β-CD-polymer NTB group compared to the Untreated (Bleo) group, suggesting enhanced modulation of specific inflammatory markers with the β-CD-polymer NTB treatment. Data represent mean ± SEM, with statistical significance indicated as *p < 0.05, **p < 0.01, and ns denoting non-significance. Multiple comparisons ANOVA was performed for each condition

Our findings highlight three important benefits of using NTB-loaded β-CD-polymer MPs—(1) reduced dosage and frequency of administration, with NTB-loaded β-CD-polymer MPs (8 mg/kg) administered only twice during the study compared to twice-daily bolus injections of NTB (30 mg/kg) throughout the duration of the study, which is standard for this model [48] (2) significant reduction in systemic inflammatory responses, as indicated by decreased levels of IFNγ, LIX, IP-10, and MCP-1 in the NTB-loaded β-CD group (Fig. 8a–d, and SI-1). The four markers (IFNɣ, LIX, IP-10, and MCP-1) were statistically different and since bleomycin induces two phases: (1) the inflammatory phase, which then triggers (2) the fibrotic phase. Therefore, reducing the inflammation, reduces the onset of the fibrosis. Thus, the reductions in the inflammatory markers suggest that the sustained release of NTB effectively alleviates inflammation-driven fibrosis. Notably, this effect was achieved with a lower NTB dose (8 mg/kg) compared to the bolus group (30 mg/kg), which showed only modest cytokine suppression, consistent with findings from Pan et al. [49] Although the broader cytokine panel (SI-1) reflects a general modulation of inflammation, the reduction of IL-9 by both NTB-loaded β-CD and bolus NTB (*p < 0.001) further supports antifibrotic activity, as IL-9 is associated with Th9 cell–driven fibroblast activation and collagen synthesis in lung fibrosis models [50–52]. Additionally, (3) both NTB treatment groups also exhibited less body weight loss compared to the untreated bleomycin group (Figs. 5b, 6, and 7k), suggesting an overall mitigation of disease burden. Although sustained-release systems are often expected to outperform bolus dosing, our data show that the β-CD NTB group achieved comparable therapeutic outcomes with fewer injections and a lower total drug dose. Matching weight loss outcomes (Fig. 6) while also reducing pro-inflammatory cytokines compared to bolus administration (see IFNɣ and LIX in Fig. 8) highlights the advantages of this sustained-release approach. These findings suggest that β-CD-polymer MPs could offer a targeted and sustained delivery of NTB, potentially leading to effective modulation of specific inflammatory pathways, better overall management of IPF, and reduction of the likelihood of infections from opportunistic diseases like TB for which IPF is a risk factor [6, 53].

Overall, our results provide a proof of concept of the potential of β-CD-polymer MPs as an effective delivery system for NTB. This would represent a significant advancement in the treatment of IPF. By extending the duration of drug release, we can improve its bioavailability and reduce the frequency of administration, addressing key limitations of current IPF therapies. Additionally, this work opens the possibility of development of combination therapies that could target multiple fibrotic pathways simultaneously including through co-delivery of multiple drugs within the same microparticle system. Future studies will focus on optimizing β-CD-polymer MPs to enhance their long-term effectiveness and better understand the mechanisms by which they modulate inflammatory pathways in IPF.

In conclusion, this study investigated a novel approach for delivering the antifibrotic drugs NTB and PFD using β-CD-polymer MPs made from an epichlorohydrin-cyclodextrin polymer. The β-CD-polymer MPs loaded with NTB extended the drug’s bioavailability, providing sustained release for up to 4 days in a healthy murine model. In a bleomycin-induced model of IPF, the NTB-loaded β-CD MPs demonstrated therapeutic efficacy by reducing levels of the pro-inflammatory cytokine IFNɣ and chemokine LIX, as well as lowering levels of the chemokines IP-10 and MCP-1. Notably, only 2 intraperitoneal injection administrations were required over a period of 7 days. Furthermore, our results also showed that intraperitoneal administration of β-CD MPs with and without drug loaded, for 15 days, did not cause any observable toxic effects in a healthy murine model thus confirming the safety of the β-CD MPs as a drug delivery vehicle (Fig. 7, and SI-2). These findings provide proof of concept for using β-CD-polymer MPs as an effective vehicle for the sustained delivery of antifibrotic agents like NTB, enabling a lower frequency of administration and reduced drug usage for the treatment of IPF.

## Supplementary Information

Below is the link to the electronic supplementary material.

Supplementary file1 (PDF 1375 KB)

## Data Availability

The data are available from the corresponding authors upon reasonable request. The rest of the results from the multiplex ELISA are available in the supplementary Information file.
