# Supplementary material for: Sustained Release of Antifibrotic Nintedanib from Polymer Microparticles Reduces Dosing Frequency While Reducing Inflammation in Murine Idiopathic Pulmonary Fibrosis
Source: Ann Biomed Eng. 2025 Apr 10;53(7):1590–603. doi: 10.1007/s10439-025-03729-8 (PMC12185609; doi:10.1007/s10439-025-03729-8)
Supplement: Supplementary file 1 — Supplementary file1 (PDF 1375 KB) [file 10439_2025_3729_MOESM1_ESM.pdf]

Sustained release of antifibrotic Nintedanib from polymer microparticles reduces dosing frequency while reducing inflammation in murine idiopathic pulmonary fibrosis

Emmanuel Einyat Opolot [a], Filip Goshevski [b], Rahul Chaudhary [b], Jessica A. Kilgore [c], Noelle S. Williams [c], Horst A. von Recum [a, #, *], and Amar B. Desai [b, #, *]

[a] Department of Biomedical Engineering, Case Western Reserve University, Cleveland, OH, USA
[b] Department of Medicine and Case Comprehensive Cancer Center, Case Western Reserve University, Cleveland, OH, USA
[c] Department of Biochemistry, University of Texas Southwestern Medical Center, Dallas, Texas, USA
[#] Corresponding authors: abd10@case.edu | hav1@case.edu
[*] Authors provided equivalent contribution to this work

Supplementary Information

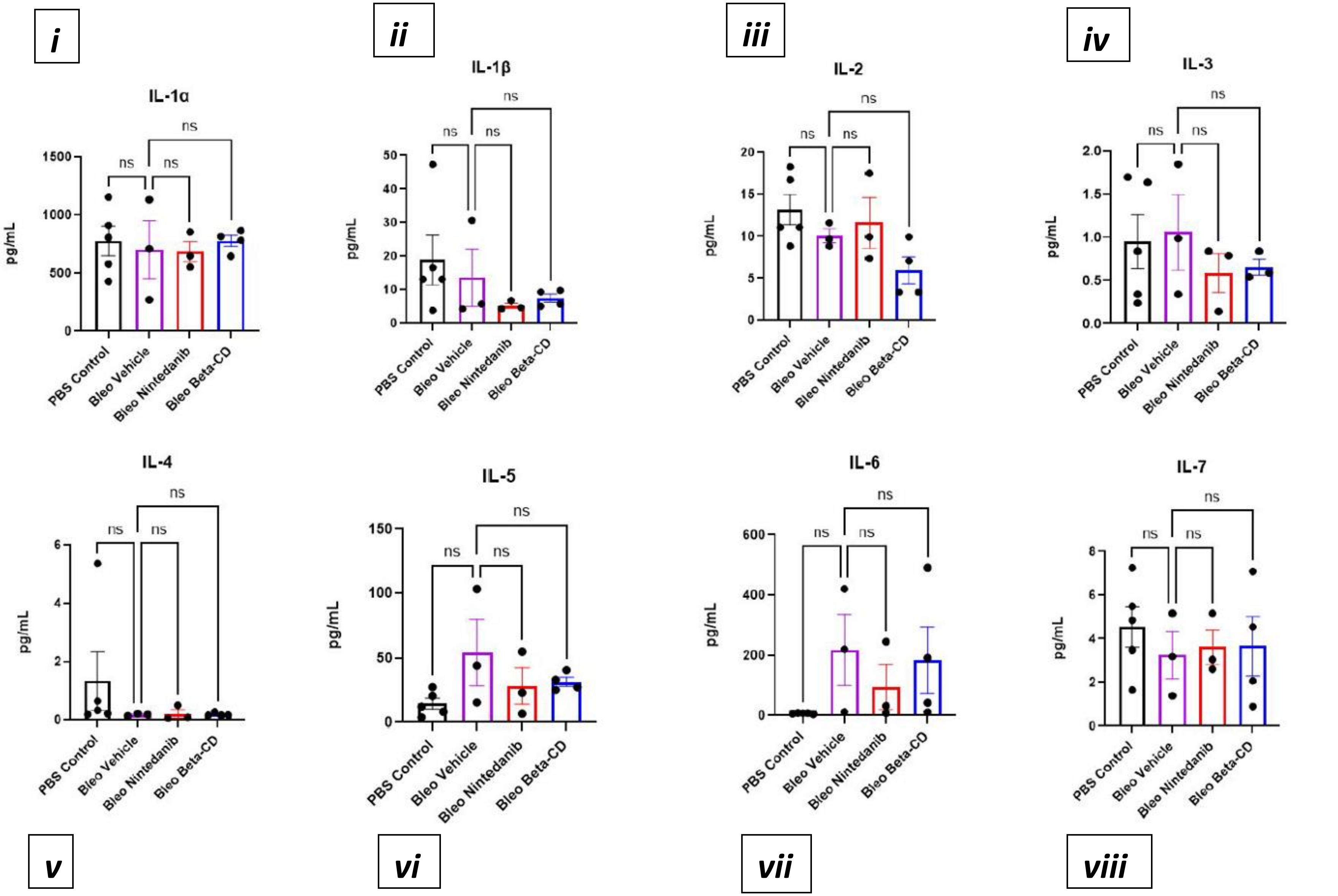

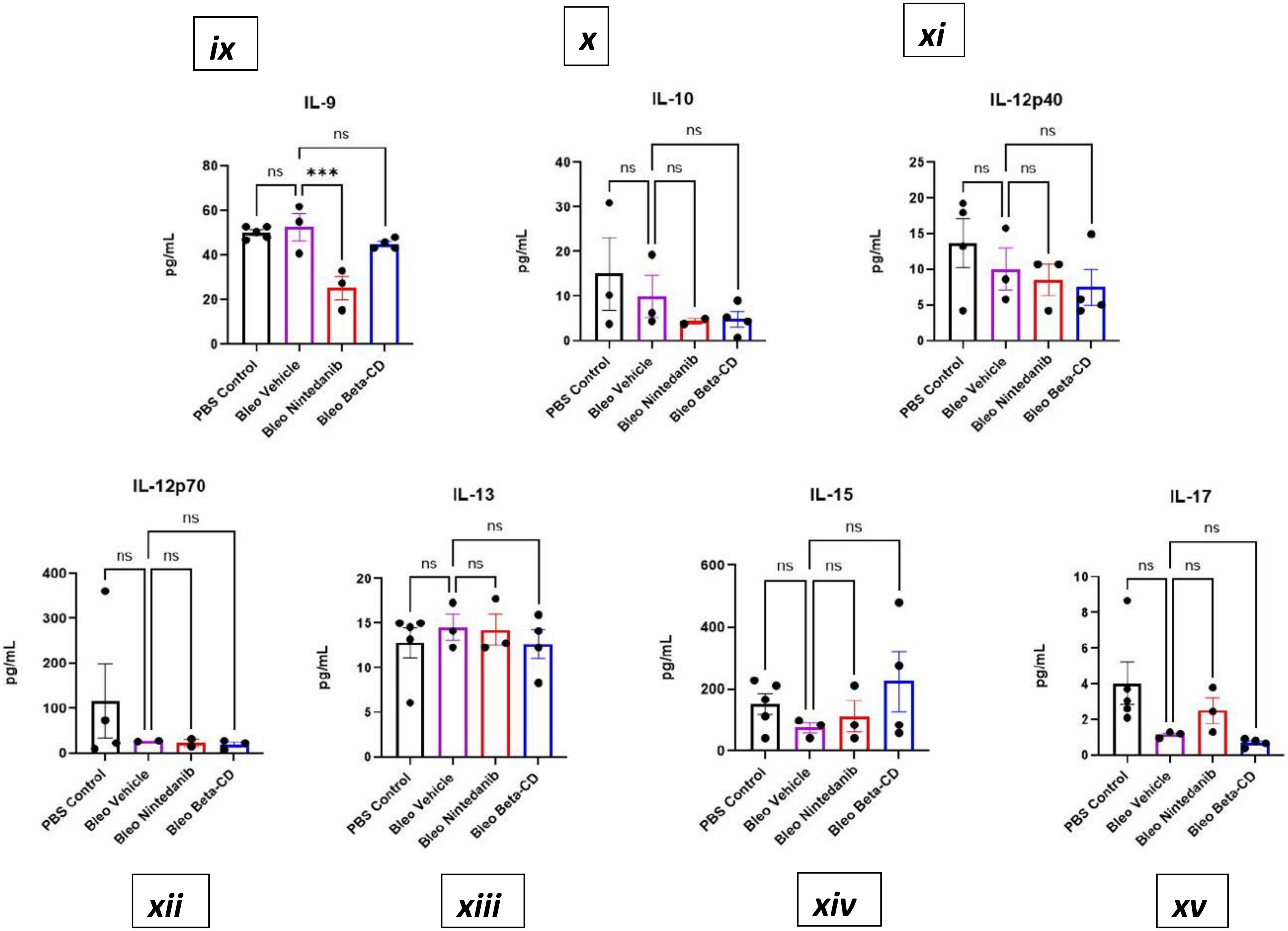
ix
IL-9
pg/mL
0
20
40
60
80
ns
***
ns
PBS Control
Bleo Vehicle
Bleo Nintedanib
Bleo Beta-CD
x
IL-10
pg/mL
0
10
20
30
40
ns
ns
ns
PBS Control
Bleo Vehicle
Bleo Nintedanib
Bleo Beta-CD
xi
IL-12p40
pg/mL
0
5
10
15
20
25
ns
ns
ns
PBS Control
Bleo Vehicle
Bleo Nintedanib
Bleo Beta-CD
IL-12p70
pg/mL
0
100
200
300
400
ns
ns
ns
PBS Control
Bleo Vehicle
Bleo Nintedanib
Bleo Beta-CD
xii
IL-13
pg/mL
0
5
10
15
20
ns
ns
ns
PBS Control
Bleo Vehicle
Bleo Nintedanib
Bleo Beta-CD
xiii
IL-15
pg/mL
0
200
400
600
ns
ns
ns
PBS Control
Bleo Vehicle
Bleo Nintedanib
Bleo Beta-CD
xiv
IL-17
pg/mL
0
2
4
6
8
10
ns
ns
ns
PBS Control
Bleo Vehicle
Bleo Nintedanib
Bleo Beta-CD
xv

xvi

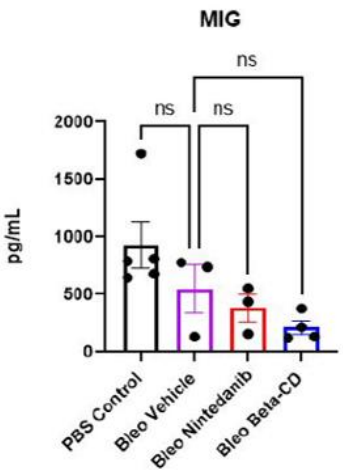

xvii

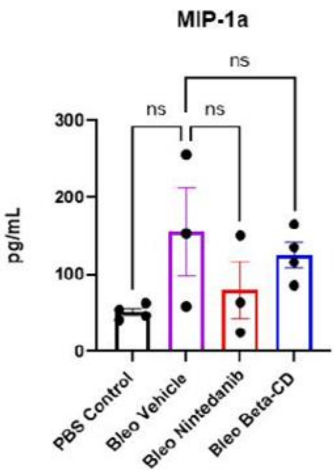

xviii

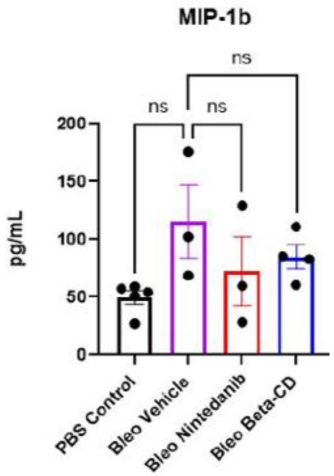

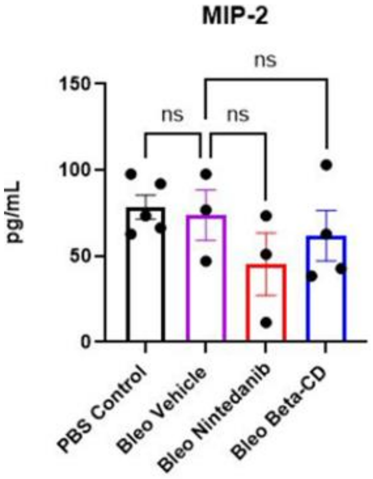

xix

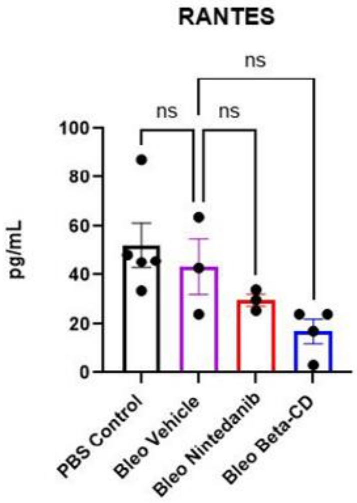

xx

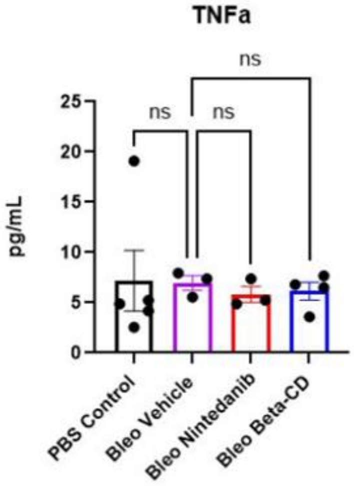

xxi

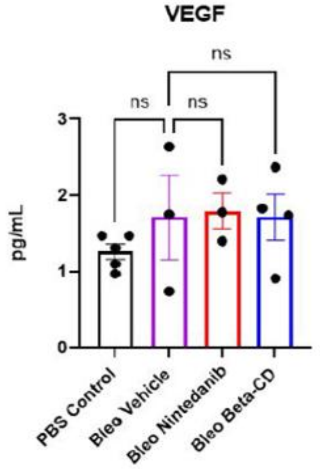

xxii

xxiii

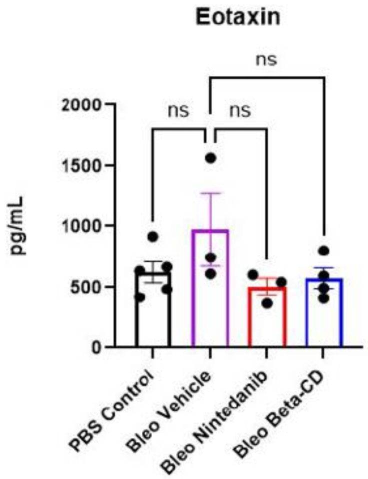

xxiv

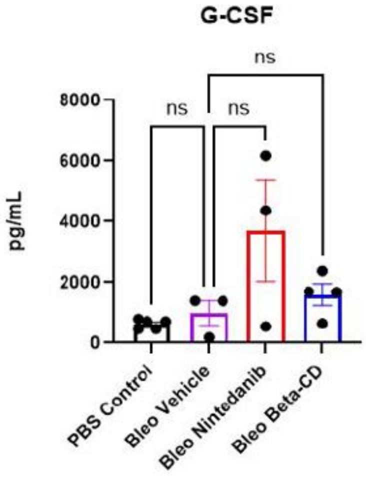

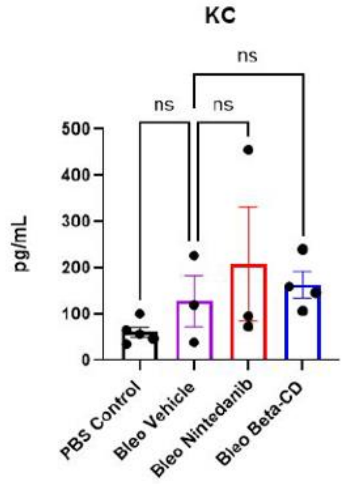

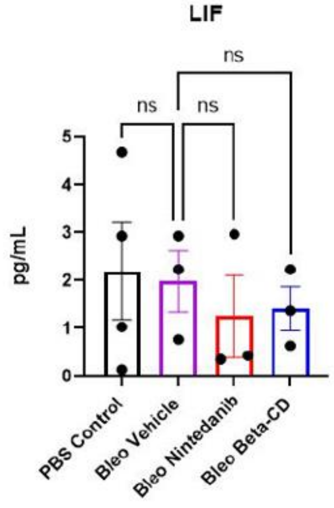

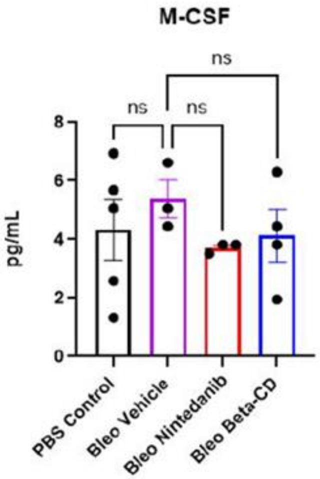

xxv

xxvi

xxvi

SI-1: Impact of Nintedanib and Beta-Cyclodextrin Microparticle Delivery on Pro-Inflammatory Cytokine Levels in bleomycin-induced Idiopathic Pulmonary Fibrosis; *(i – viii)* levels of pro-inflammatory cytokines, including IL-1α, IL-1β, IL-2, IL-3, IL-4, IL-5, IL-6, and IL-7, were measured in the blood serum of bleomycin-induced pulmonary fibrosis mouse models following treatment with either free Nintedanib (Bleo + Nintedanib) or Nintedanib loaded into Beta-Cyclodextrin microparticles (Bleo + Beta-CD). Data were collected over 7 days via multiplex ELISA. No significant differences (ns) were observed between the groups in the measured cytokine levels, suggesting no exacerbation of inflammatory responses when utilizing the beta-cyclodextrin microparticle delivery system compared to the free drug. ***(ix – xv)*** Cytokines associated with anti-inflammatory and regulatory functions, such as IL-9, IL-10, IL-12p40, IL-12p70, IL-13, IL-15, and IL-17, were analyzed in the serum of bleomycin-treated mice. Notably, IL-9 levels were significantly reduced (***p < 0.001) in the Bleo Nintedanib group, and low in Nintedanib-loaded Beta-Cyclodextrin group compared to the Bleo vehicle, while other cytokines showed no significant differences between the treatment groups. These results indicate that Beta-Cyclodextrin may influence certain inflammatory pathways differently than free Nintedanib. ***(xvi – xxii)*** Chemokine levels, including Eotaxin, G-CSF, KC, LIF, and M-CSF, were measured to evaluate the potential impact on neutrophil and macrophage recruitment in response to treatment. Although no statistically significant differences (ns) were observed across the treatment groups, the data provide insight into how Nintedanib and its microparticle formulation may alter the chemotactic environment in bleomycin-induced pulmonary fibrosis. ***(xxiii – xxvi)*** Multiplex ELISA analysis was used to evaluate several chemokines and growth factors (MIG, MIP-1α, MIP-1β, MIP-2, RANTES, TNFα, and VEGF) in the blood serum of mice following intraperitoneal administration of free Nintedanib or Nintedanib-loaded Beta-Cyclodextrin microparticles. Despite the different delivery mechanisms, no significant differences (ns) were noted in the expression of these factors, suggesting comparable modulation of these mediators by both treatment strategies. Each data point represents an individual mouse (n = 5). Bars represent mean ± SD.

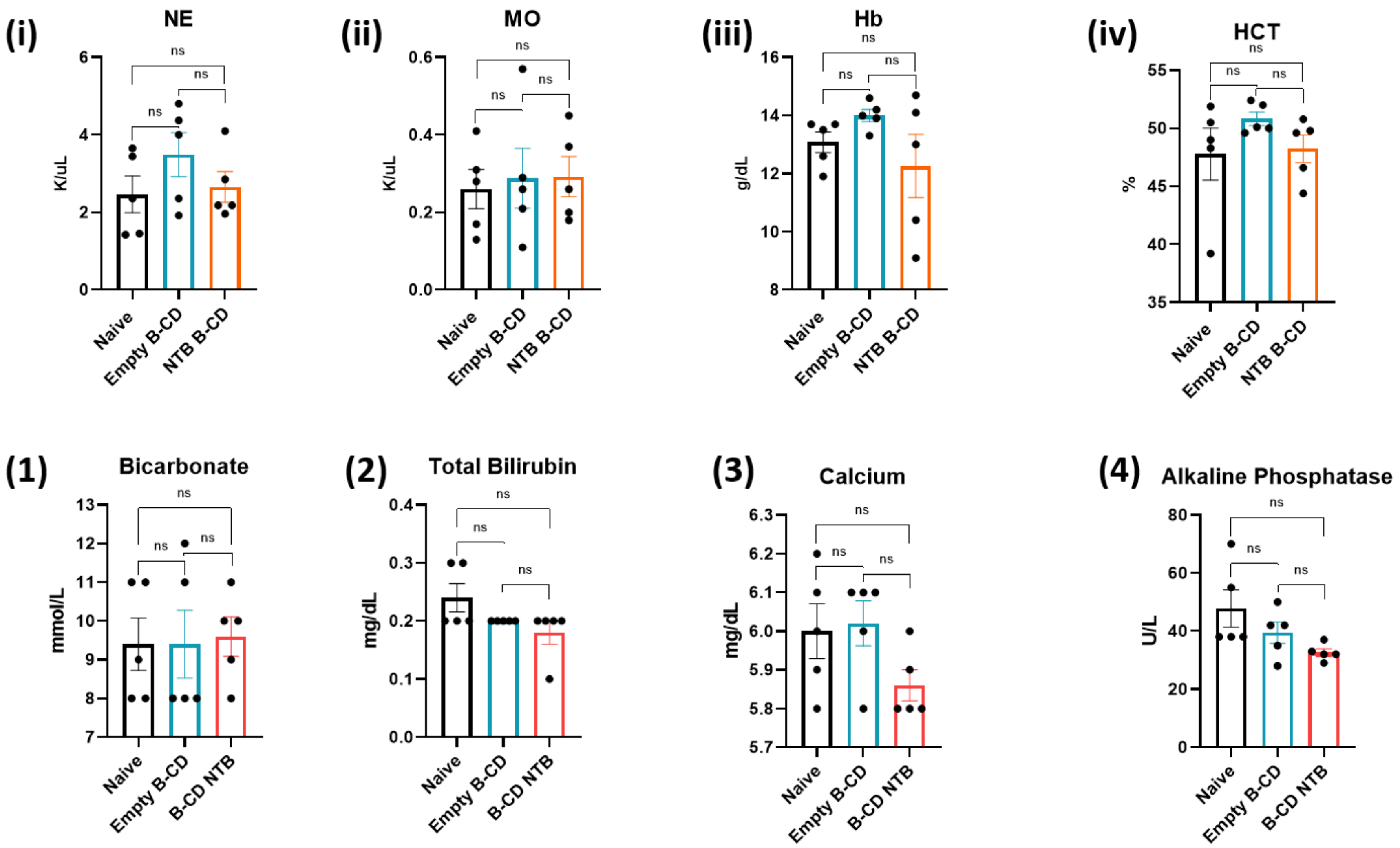

SI-2: Hematological and serum biochemical analysis following treatment with B-CD formulations. Hematological parameters including (i) neutrophil count (NE), (ii) monocyte count (MO), (iii) hemoglobin levels (Hb), and (iv) hematocrit (HCT) were assessed in mice treated with naïve control, empty B-CD, or NTB B-CD. Additionally, serum biochemical markers including (1) bicarbonate, (2) total bilirubin, (3) calcium, and (4) alkaline phosphatase were measured across treatment groups. Data are presented as mean ± SEM (n = 5 per group). Statistical comparisons were performed using one-way ANOVA with multiple comparisons, with "ns" denoting no significant

differences between groups. This is supporting data for the hematology assessment and the biochemical analysis done to investigate the toxicity of administration of B-CD microparticles with or without drug loaded. No significant differences were seen or observed from the Empty B-CD or the B-CD NTB treatment groups.
